# Role of heparanase in pulmonary hypertension

**DOI:** 10.3389/fphar.2023.1202676

**Published:** 2023-08-11

**Authors:** Lin-Jun Wang, Fei Feng, Jian-Chun Li, Ting-Ting Chen, Li-Ping Liu

**Affiliations:** ^1^ The First Clinical Medical School of Lanzhou University, Lanzhou, Gansu, China; ^2^ Departments of Emergency Critical Care Medicine, The First Hospital of Lanzhou University, Lanzhou, Gansu, China

**Keywords:** heparanase, pulmonary hypertension, vascular endothelial cell, inflammation, coagulation, glycocalyx

## Abstract

Pulmonary hypertension (PH) is a pathophysiological condition of increased pulmonary circulation vascular resistance due to various reasons, which mainly leads to right heart dysfunction and even death, especially in critically ill patients. Although drug interventions have shown some efficacy in improving the hemodynamics of PH patients, the mortality rate remains high. Hence, the identification of new targets and treatment strategies for PH is imperative. Heparanase (HPA) is an enzyme that specifically cleaves the heparan sulfate (HS) side chains in the extracellular matrix, playing critical roles in inflammation and tumorigenesis. Recent studies have indicated a close association between HPA and PH, suggesting HPA as a potential therapeutic target. This review examines the involvement of HPA in PH pathogenesis, including its effects on endothelial cells, inflammation, and coagulation. Furthermore, HPA may serve as a biomarker for diagnosing PH, and the development of HPA inhibitors holds promise as a targeted therapy for PH treatment.

## 1 Introduction

Pulmonary hypertension (PH) is characterized by a mean pulmonary arterial pressure (mPAP) ≥25 mmHg at rest, although recent studies have suggested that an upper limit of 20 mmHg should be considered normal (Simonneau et al., 2019). Despite the increasing global research efforts on PH, significant breakthroughs in its pathogenesis are still lacking, and the 5-year mortality rate remains at approximately 50% (Thenappan et al., 2018). Increased pulmonary vascular pressure disrupts hemodynamic balance, leading to elevated right ventricular afterload, right heart failure, and potentially fatal outcomes (Cassady and Ramani, 2020). The right ventricle possesses thin ventricular walls and good compliance, but its structural characteristics make it less tolerant to pressure changes. Unfortunately, clinical attention to right heart failure is often overshadowed by the focus on left heart failure, potentially resulting in deteriorating conditions for patients, particularly those who are critically ill. Recognizing the significance of right heart function, the American Heart Association issued “Assessment and Management of Right Heart Failure” in 2018, emphasizing the importance of addressing this aspect of cardiac health (Konstam et al., 2018).

**FIGURE 1:**
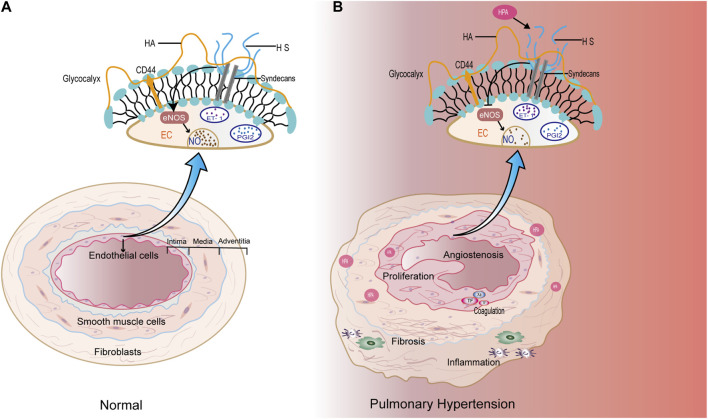
**(A)** Under physiological conditions, endothelial cells maintain normal functions through various components, and the pulmonary vascular structures are normal. **(B)** In the pathological condition of PH, high expression of HPA specifically degrades HS, affecting the production of NO and endothelin-1 (ET-1) in pulmonary artery endothelial cells. In addition, HPA may promote pulmonary artery proliferation, which is closely related to inflammation, coagulation, and fibrosis.

The current classification of PH aligns with the 2022 guidelines from the European Society of Cardiology (ESC) and the European Respiratory Society (ERS) (Humbert et al., 2022). It categorizes PH into five main groups: 1. pulmonary arterial hypertension; 2. PH due to left heart disease; 3. PH due to lung diseases and/or hypoxia; 4. chronic thromboembolic PH and other pulmonary artery obstructions; and 5. PH with unclear and/or multifactorial mechanisms. The etiology of PH is complex and involves various factors, including hypoxia, inflammation, genetics, drug-related causes, thrombosis, and left heart disease (Galiè et al., 2015). Remodeling of the pulmonary arteriole vessels and proliferation of the pulmonary artery smooth muscle layer are characteristic features of PH (Poch and Mandel, 2021). Endothelial dysfunction plays a significant role in the development of PH, involving pathways such as nitric oxide (NO), endothelin, and prostaglandin (PGI2) (Del Pozo et al., 2017; Lázár et al., 2020; Zhang and Xu, 2020) (Figure 1A). Additionally, inflammatory reactions, immune responses, and coagulation abnormalities contribute to the pathological mechanism of PH. Pulmonary vascular fibrosis has been shown to promote the progression of PH (Zhang et al., 2020a) (Figure 1B). Currently, the diagnosis of PH still relies on invasive procedures, such as right heart catheterization, or non-invasive techniques, like ultrasound examinations. However, there is a lack of reliable experimental markers or biomarkers to aid in the diagnosis of PH. Treatment options for PH include drugs targeting the NO, prostacyclin, and endothelin pathways, which help improve hemodynamics in PH patients. However, the long-term prognosis for PH patients remains poor (Zolty, 2020). Therefore, ongoing research in the field of PH focuses on exploring the underlying pathogenesis, identifying diagnostic biomarkers, and developing targeted therapeutic drugs to improve patient outcomes.

Heparanase (HPA) is the sole endoglycosidase capable of degrading heparan sulfate (HS) in the extracellular matrix (Rivara et al., 2016). Research on HPA has primarily focused on inflammation and tumor metastasis, and HPA also plays a crucial role in the coagulation system (Nadir, 2020). Additionally, HPA has been implicated in tissue fibrosis, angiogenesis, and cell proliferation (Lv et al., 2016; Lv et al., 2018). Recent studies suggest that HPA may be involved in the pathophysiological mechanisms of PH. A search using relevant terms, such as “pulmonary hypertension, heparanase, vascular endothelial cells, inflammation, coagulation, glycocalyx, autophagy, exosomes, and fibrosis,” in databases like PubMed and Web of Science reveals the involvement of HPA in pulmonary blood vessels. For instance, HPA promotes the adhesion of neutrophils to the vascular endothelium and the degradation of the pulmonary endothelial layer (Schmidt et al., 2012). HPA can degrade HS in the glycocalyx, which is present in the pulmonary artery, and inhibitors of HPA have been shown to reduce pulmonary artery pressure (Guo et al., 2019). Moreover, HS plays a vital role in activating endothelial nitric oxide synthase (eNOS) in pulmonary vascular endothelial cells, thereby reducing pulmonary vascular permeability (Dull et al., 2012). These findings indicate that HPA may participate in the development of PH through its involvement with endothelial cells, inflammation, coagulation, and fibrosis (Figure 1B). However, reports specifically linking HPA to PH are limited. Therefore, this review represents the first attempt to elucidate the relationship between HPA and PH through various pathways.

## 2 Biological structure and characteristics of HPA

### 2.1 Discovery and basic structure of HPA

HPA, an endo-β-glucuronidase, is responsible for cleaving HS polysaccharide chains. Its enzymatic activity was initially reported in 1975 (Höök et al., 1975). Subsequently, five research groups isolated 50-kDa HPA from the human placenta and identified the HPA gene in the human genomic DNA library. The HPA gene is located on human chromosome 4q22, spanning approximately 50 kb and consisting of 14 exons and 13 introns (Vlodavsky et al., 1999). Human HPA comprises an α (β/α)8 domain and an αβ three-dimensional domain (Yuan et al., 2022). In the three-dimensional structure of HPA, the C-terminal region plays a critical role in its enzymatic activity and secretion (Cruz et al., 2020). The primary function of HPA is the degradation of HS in the extracellular matrix, which contributes to processes such as tumor metastasis and inflammation (Nadir, 2020; Zhu et al., 2020). Additionally, HPA exhibits non-enzymatic activity. Studies have shown that HPA can facilitate primary tumor progression independently of its enzymatic activity (Yang et al., 2022).

### 2.2 The function of HPA

Currently, three main types of HPA have been identified: HPA I, HPA II, and HPA III. Each of these HPAs possesses a leader sequence that determines their substrate specificity. HPA I primarily cleaves the anti-GlcNS3S6S-IdoA2S chain. IdoA2S is present in the binding site of thrombin III and the AT-binding site of heparin, making heparin the specific substrate for HPA I (Xiao et al., 2011). On the other hand, HPA III displays a preference for the HS domain and has a unique substrate specificity for HS synthesis (Hu et al., 2017). HPA II has a relatively broad range of substrates, including both heparin and HS (Vlodavsky et al., 2018). In a recent study, alternative splicing of the HPA 2 gene resulted in the encoding of three proteins: HPA 2a, HPA 2b, and HPA 2c. Notably, the HPA 2c protein acts as an inhibitor of HPA activity, suggesting that HPA 2 may be associated with a favorable prognosis in head and neck carcinoma (Levy-Adam et al., 2010). Among the different HPAs, HPA I has been the most extensively researched and utilized. However, the application of HPA is often hindered due to its poor thermal stability (Chen et al., 2011).

### 2.3 The regulation of expression of HPA

**FIGURE 2:**
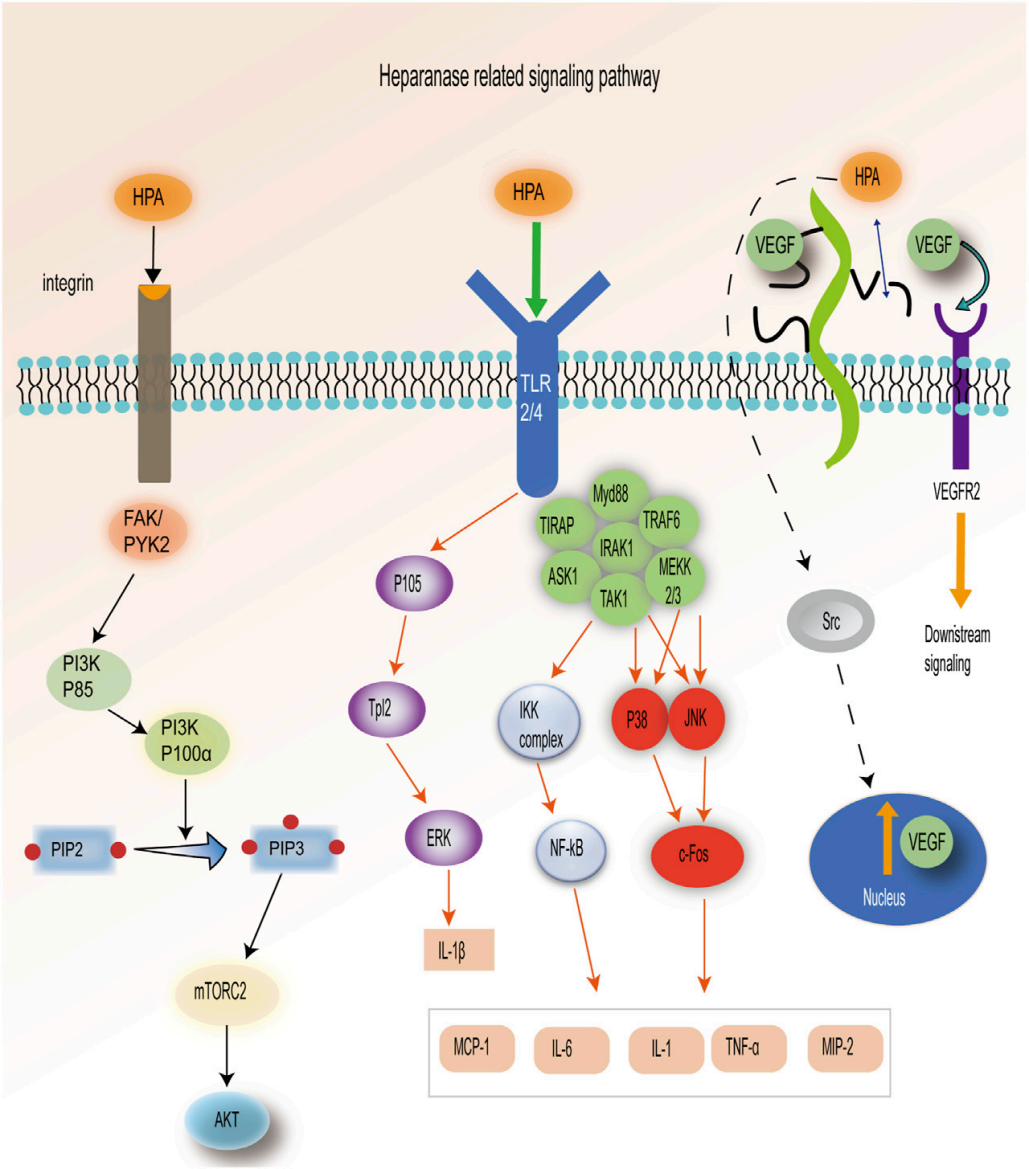
HPA-related signal pathways: 1. HPA is dependent on the PI3K–mTORC2–Akt pathway. 2. HPA activates ERK, p38, and JNK pathways by stimulating TLR2/4. 3. HPA plays a role in releasing VEGF through the Src pathway and degradation of HS. [Adapted from Koganti et al. (2020)].

Under physiological conditions, HPA is present in specific normal tissue cells, including keratinocytes, trophoblast cells, platelets, mast cells, white blood cells, and capillary endothelial cells (Vlodavsky et al., 1999). However, under pathological conditions, elevated expression of HPA promotes angiogenesis and inflammation in malignant tumors (Lindahl and Li, 2020). The expression of the HPA gene is regulated by various factors, including transcription factors, cytokines, growth factors, and other signaling molecules. HPA is a versatile protein with both enzymatic and non-enzymatic functions, which trigger multiple signaling pathways, such as Akt and Src (Secchi et al., 2015). First, HPA can induce Akt phosphorylation in various tumor-derived cell lines, and the activation of phosphatidylinositol 3-kinase (PI3K) is necessary for HPA-induced Akt activation (Hao et al., 2015). The activation of PI3K will convert PIP2 to PIP3, further activate mTORC2, and bind to Akt (Gan et al., 2011). HPA mediates Akt phosphorylation of Ser473 residues in a mTORC2-dependent manner. P110α was the PI3K catalytic isoform preferred by HPA for AKT activation and cell proliferation. The process requires the participation of integrins FAK and PYK2 (Riaz et al., 2013). Second, in a Gutter-Kapon study, HPA activated TLRs required for ERK, p38, and JNK signal transduction in macrophages. These three proteins continued to activate c-Fos and finally interacted with different cytokines (Gutter-Kapon et al., 2016). HPA activates TLR2/TLR4 through an unknown mechanism, which leads to the activation of P105, downstream activation of Tpl2 and ERK, and the production of IL-1β. The activation of TLR2/4 can stimulate the p38, JNK, and NF-κB signaling pathways through the formation of MyD88-dependent protein complexes, resulting in the production of TNF-α, IL-1, IL-6, TNF-α, MCP-1, and MIP2 (Koganti et al., 2020). Lastly, overexpression of HPA increases the level of vascular endothelial growth factor (VEGF) protein, which can be effectively inhibited by Src inhibitors (Zetser et al., 2006). HPA is involved in the regulation of VEGF gene expression through Src activation. Moreover, HPA can cleave HS chains from perlecan, releasing VEGF to bind to VEGFR2 and stimulate downstream signaling (Kadenhe-Chiweshe et al., 2008). Therefore, HPA participates in various signaling pathways (Figure 2).

## 3 The role of HPA in the development of PH

HPA participates in the occurrence of PH in different ways. HPA leads to endothelial cell dysfunction through the NO pathway and regulates a variety of vascular growth factors in endothelial cells. In addition, HPA also mediates the expression of various inflammatory factors (IL-6, IL-8, and TNF-α) and coagulation factors (TF, TFPI, and PLT) in PH (Table 1).

**TABLE 1:** HPA participates in PH by different factors.

Path	Related biomarker	HPA function	Expression in PH	References
HPA causes PH by affecting vascular endothelial cells	VEGF bFGF	Promoted	Increased	38, 56
	Ang-2	Promoted	Increased	52, 58
	No	Promoted inhibition	Increased/decreased	53, 57, 48, 55
HPA causes PH by affecting inflammation factors	TNF-α	Promoted	Increased	17, 69
	IL-1	Promoted	Increased	63, 71
	IL-6	Promoted	Increased	64, 70
	IL-8	Promoted		65, 71
HPA causes PH by affecting coagulation factors	TF	Promoted	Increased	74, 84
	TFPI	Dissociated	Decreased/increased	77, 85
	PLT	Promoted		81, 86

### 3.1 HPA participates in PH through affecting vascular endothelial cells

Vascular endothelial cells form the inner layer of blood vessels and play crucial roles in inflammation, neovascularization, and vasoconstriction. The surface of vascular endothelial cells is covered by a glycosaminoglycan (GAGs) polysaccharide–protein complex called the glycocalyx (Suzuki et al., 2022). The main components of the glycocalyx are HS, hyaluronic acid (HA), and syndecan-1 (SDC-1) (Guo et al., 2019). The glycocalyx serves as a direct barrier between blood flow and vascular endothelial cells. HS and SDC-1 are important for endothelial cell mechanotransduction and blood flow remodeling (Ebong et al., 2014). In addition, Piezo1, a mechanosensitive channel, is involved in vascular remodeling (Chen et al., 2022). Recent research has shown that Piezo1 is upregulated in lung vascular endothelial and smooth muscle cells in rats with PH, as well as in human pulmonary artery endothelial cells (PAECs) and lung tissues (Wang et al., 2021). Interestingly, retrograde perfusion of the diabetic heart leads to significantly higher levels of HPA release, which may be attributed to an increase in Piezo1 expression (Lee et al., 2022). Therefore, HPA may participate in the development of PH through Piezo1-mediated mechanical sensing. The NO pathway is one of the most common pathways associated with endothelial cell dysfunction. The production of NO relies on the integrity of the glycocalyx (Bush et al., 2021). Research has shown that the glycocalyx participates in the mechanosensing and transduction of endothelial cells by activating endothelial nitric oxide synthase (eNOS) through its HS component (Yen et al., 2015). HS can activate endothelial cells to produce NOS (Lucena et al., 2018). HPA III, when used to degrade HS, impairs NO production in bovine aortic endothelial cells (Florian et al., 2003). Therefore, the specific degradation of HS by HPA leads to a decrease in eNOS activity and NO production, ultimately affecting endothelial cell function. Interestingly, the glycocalyx of endothelial cells sheds in COVID-19 patients, and heparin has been shown to attenuate glycocalyx shedding (Potje et al., 2021). In diabetes, activation of the endothelin-1 signaling pathway induces HPA expression in podocytes and damages the glycocalyx (Garsen et al., 2016). High expression of HPA is associated with the endothelin A receptor in epithelial ovarian cancer (Anggorowati et al., 2018). Furthermore, HPA regulates the expression of the VEGF, basic fibroblast growth factor (bFGF), and angiopoietin (Ang) in vascular endothelial cells (Kadenhe-Chiweshe et al., 2008; Li et al., 2012; Zhang et al., 2013). Ang-2 stimulates endothelial cells to release HPA (Lukasz et al., 2017).

Endothelial dysfunction is indeed a critical factor in the development of PH, particularly in relation to the NO pathway (Evans et al., 2021). Various vascular regulatory factors in endothelial cells also play crucial roles in PH. For instance, elevated levels of VEGF have been associated with vascular smooth muscle cell proliferation in a mouse model of hypoxic PH (Liu et al., 2018). High concentrations of Ang-2 and bFGF have been identified as significant poor prognostic factors in PH (Seyfarth et al., 2015; Enomoto et al., 2021). It can be seen that there is a close relationship between HPA and pulmonary artery endothelial cells. However, endothelial dysfunction is a necessary condition for the occurrence of PH. Therefore, HPA may play an important role in PH by regulating the function of vascular endothelial cells.

### 3.2 HPA participates in PH by affecting inflammation

Inflammation is a complex process that plays a significant role in various diseases and involves multiple cell types. The role of HPA in inflammation has been extensively studied in conditions such as sepsis (Liao et al., 2023), acute respiratory distress syndrome (ARDS) (Feng et al., 2023), chronic colitis (Lerner et al., 2011), and other inflammatory diseases. Schmidt et al. (2012) demonstrated that HPA promotes neutrophil aggregation and degradation of the glycocalyx through the TNF-α pathway in a mouse model of sepsis. HPA inhibitors have been shown to possess anti-inflammatory effects (Xiang et al., 2022). Increased HPA expression in the abdominal cavity of mice enhances the inflammatory response by elevating levels of TNF-α and IL-1 (Blich et al., 2013). HPA is involved in macrophage activation, leading to increased production of pro-inflammatory cytokines, such as TNF-α, IL-6, and IL-1β (Goodall et al., 2014). Moreover, HPA promotes the expression of IL-6 and IL-8 in acute renal injury, and inhibition of HPA attenuates the inflammatory response (Abassi and Goligorsky, 2020). HPA inhibitors have been shown to reduce the overexpression of IL-1 in septic mice (Fu et al., 2022). HPA contributes to the inflammatory process through interactions with various inflammatory factors. Furthermore, HPA’s non-enzymatic activity promotes inflammatory cell adhesion and the inflammatory response (Vlodavsky et al., 2016). The involvement of HPA in inflammation is complex and depends on factors such as the specific cell types involved and the nature of the inflammatory response (Stoler-Barak et al., 2015).

Vascular response is a central component of the inflammatory process, and inflammation is known to play a critical role in the pathogenesis of PH. TNF-α, for example, can activate the ALK2/ACTR-IIA signaling axis and induce the proliferation of pulmonary artery smooth muscle cells (PASMs) (Hurst et al., 2017). Numerous inflammatory factors have been implicated in the development of PH, including increased expression of IL-1, IL-6, and IL-8 (Pandolfi et al., 2017; Udjus et al., 2019). PH is characterized by the presence of abundant macrophages, lymphocyte infiltration, and significantly elevated levels of inflammatory factors, such as IL-1, IL-6, and TNF-α, which collectively regulate the proliferation and apoptosis of PASMs (Soon et al., 2010). In PH patients, the reduced levels of HS contribute to increased inflammatory cell extravasation and potentially lead to pathological vascular remodeling (Biasin et al., 2018). In summary, HPA can promote inflammatory responses, and inflammation plays a crucial role in the proliferation of PASMs and the development of PH. Therefore, HPA may play an important role in PH by activating inflammatory processes.

### 3.3 HPA participates in PH by affecting the coagulation function

HPA is closely related to the coagulation cascade reaction, and heparin is one of the substrates of HPA. Studies have shown that HPA can induce tissue factor (TF), promoting coagulation activity through the phosphorylation of the p38 pathway (Nadir and Brenner, 2010). Inhibition of HPA reduces TF overexpression in septic mice (Fu et al., 2022). HPA enhances Xa activity by promoting TF and activates the coagulation pathway (Peled et al., 2016). In mouse arterial injury models, overexpression of HPA leads to the formation of larger thrombi in a relatively short period of time (Baker et al., 2012). The tissue factor pathway inhibitor (TFPI) is a plasma serine protease inhibitor that plays a crucial role in maintaining balance and regulation in the coagulation system. High expression of HPA leads to the release of TFPI from the cell surface (Crispel et al., 2017). HPA upregulates TF expression and interacts with TFPI on the cell surface membrane, resulting in increased coagulation activity (Nadir, 2014). In Cui et al. (2016), increased HPA expression in a mouse model enhanced platelet activity, promoting blood coagulation and thrombosis. Furthermore, recent reports have suggested that the non-enzymatic activity of HPA contributes to its procoagulant function as HPA can directly activate Xa activity, promoting coagulation (Nadir and Brenner, 2012). Anti-HPA therapy has been shown to inhibit platelet activation (Yang et al., 2020).

Indeed, pulmonary artery thrombosis is a significant factor in the pathogenesis of PH, and the coagulation reaction plays a central role in thrombus formation. Studies have confirmed the presence of hypercoagulability in patients with idiopathic pulmonary hypertension (IPH) (Tournier et al., 2010). TF, as the promoter of the exogenous coagulation pathway, plays an important role in PH by promoting vascular remodeling (Deng et al., 2016). Abnormal expression of TF triggers the proliferation of smooth muscle cells and leads to thrombosis in the vascular cavity (Cimmino and Cirillo, 2018). Reduced expression of the tissue factor pathway inhibitor (TFPI) is observed in hypoxic PH mice, and TFPI has been shown to inhibit pulmonary vascular remodeling (White et al., 2010). Furthermore, platelets can contribute to pulmonary vascular constriction and promote abnormal angiogenesis, leading to the development of neonatal PH (Davizon-Castillo et al., 2020). In summary, HPA plays a significant role in coagulation, and the coagulation reaction not only contributes to the pathogenesis of thrombotic PH but also participates in the hypercoagulable state and angiogenesis observed in PH. Therefore, HPA may contribute to increased pulmonary artery pressure through its involvement in the coagulation process.

### 3.4 HPA participates in PH by affecting fibrosis

Fibrosis is a significant factor in tissue repair and can lead to tissue structural damage and organ dysfunction when it becomes excessive or persistent (Henderson et al., 2020). HPA has been shown to influence tissue fibrosis, thereby contributing to organ dysfunction. Studies have demonstrated that a high expression of HPA induces chronic fibrosis in mouse liver injuries, while HPA inhibitors decrease the expression of alpha-smooth muscle actin (α-SMA) and alleviate liver fibrosis (Lv et al., 2016). HPA is also involved in promoting fibrosis in the lungs, kidneys, and other organs. The development of pulmonary artery hyperplasia and fibrosis is a fundamental condition for the occurrence of PH. Studies have shown increased expression of α-SMA in mouse models of PH, and microRNA150 has been found to protect against hypoxia-induced pulmonary vascular fibrosis, leading to a reduction in pulmonary artery pressure (Li et al., 2019). Inhibitors of galectin-3, a protein involved in fibrosis, have been shown to attenuate and reverse pulmonary artery remodeling, fibrosis, and hemodynamic indices in rat models of PH (Barman et al., 2019). Transforming growth factor-α (TGF-α), which plays a crucial role in promoting organ fibrosis through both Smad-dependent and non-Smad-dependent pathways, has been implicated in the development of pulmonary fibrosis and significant PH (Frangogiannis, 2020; Zhang et al., 2022). Additionally, TGF-β1 has been shown to induce pulmonary fibrosis and endothelial cell apoptosis, leading to the development of PH (Bellaye et al., 2018; Lei et al., 2022). Furthermore, HS has been identified as a mediator for the targeted delivery of TGF-β1 binding peptides to the liver, inhibiting TGF-β1 activity and improving liver fibrosis (Ding et al., 2022). HPA has been found to promote endothelial cell fibrosis, and inhibiting HPA can significantly reduce TGF-α expression in endothelial cells, thus alleviating fibrosis (Masola et al., 2017).

In summary, HPA plays a significant role in the pathogenesis of PH by affecting pulmonary endothelial function, inflammation, coagulation, and fibrosis. The updated definition of pre-capillary PH by the 6th World Symposium on Pulmonary Hypertension (WSPH) considers hemodynamic parameters, such as mPAP > 20 mmHg, PAWP ≤ 15 mmHg, and PVR ≥ 3WU (Simonneau et al., 2019). Specific targeted therapies, such as prostacyclin, have been shown to improve the prognosis of patients with pre-capillary PH (Waxman et al., 2021). This review focuses on the involvement of HPA in pre-capillary PH, including pulmonary inflammation, pulmonary artery thrombosis, and pulmonary arterial remodeling. Further research is needed to explore the relationship between HPA and post-capillary PH, particularly in PH associated with left heart disease, and to address the underlying primary diseases that contribute to PH.

## 4 The possible mechanism of HPA in PH

HPA’s involvement in autophagy, exosomes, and ferroptosis provides additional insights into its possible mechanism in PH. Autophagy, as a process of cellular self-renewal and homeostasis, has been linked to HPA. HPA can induce autophagy in inflammation and tumor cell metastasis (Sanderson et al., 2017). HPA-overexpressing tumor cells were more resistant to stress and chemotherapy in a manner associated with increased autophagy (Shteingauz et al., 2015). Exosomes, secreted vesicles involved in intercellular signaling, have also been implicated in HPA’s mechanism. HPA has been shown to activate the syndecan–syntenin–ALIX pathway of exosome biogenesis, promoting tumor progression (Thompson et al., 2013; Roucourt et al., 2015). Autophagy and exosome secretion are closely interconnected processes, further highlighting their potential relevance in PH (Vlodavsky et al., 2021). Additionally, ferroptosis, a distinct form of cell death, has emerged as a research focus in various diseases (Li et al., 2020). HPA-driven sequential released nanoparticles and ferroptosis have been studied in tumor cells, suggesting a potential role for HPA in this mechanism (Zhang et al., 2021).

The molecular mechanisms underlying PH are complex and still not fully understood. However, it is noteworthy that autophagy, exosomes, and ferroptosis have been implicated in the development of PH. Dysregulation of autophagy-related proteins, such as Beclin-1 and LC3, has been associated with pulmonary vascular remodeling in animal models of PH (Deng et al., 2016). Silencing the expression of autophagy protein LC3 and inhibiting the mTOR pathway may protect the role of PH (Mizumura et al., 2016; Chen, 2019). Exosomes derived from mesenchymal stem cells have demonstrated anti-proliferative and anti-inflammatory effects, reducing pulmonary artery pressure (Lee et al., 2012; Zhang et al., 2020b). Furthermore, exosomes derived from pulmonary artery endothelial cells have been implicated in regulating vascular fibrosis through collagen expression (Samokhin et al., 2018). The role of ferroptosis in PH has also been explored, with studies highlighting the involvement of specific molecules and pathways, such as SLC7A11 and the HMGB1/TLR4/NLRP3 inflammasome signaling pathway, in hypoxia-induced PH and inflammatory responses (Hu et al., 2022; Xie et al., 2022). Considering the involvement of HPA in autophagy, exosome biology, and ferroptosis, it is plausible to speculate that HPA may mediate the mechanisms of PH through these pathways. However, further research is needed to elucidate the specific contributions of HPA in autophagy, exosome biology, and ferroptosis to the pathogenesis of PH.

## 5 Summary

In conclusion, HPA appears to have a significant role in the pathogenesis of PH. It affects the function of pulmonary artery endothelial cells by degrading HS in the glycocalyx, leading to endothelial dysfunction and increased pulmonary artery pressure. HPA is also involved in inflammation, coagulation dysfunction, autophagy, exosomes, and fibrosis, which are all key processes associated with PH. HPA inhibitors may be a new direction to reduce the mortality of PH. In the future, HPA may be able to predict the early occurrence of PH and be used as a biomarker in PH. However, currently, there are few studies for HPA with PH, and it requires further research.

## References

[B1] Abassi Z. Goligorsky M. S. (2020). Heparanase in acute kidney injury. Adv. Exp. Med. Biol. 1221, 685–702. 10.1007/978-3-030-34521-1_28 32274732 PMC7369981

[B2] Anggorowati N. Md, P. Ghozali A. Widodo I. Sari D. C. R. Mansyur Romi M. Arfian N. (2018). Upregulation of endothelin-1/endothelin A receptor expression correlates with heparanase expression in ovarian carcinoma. Iran. J. Med. Sci. 43 (3), 286–295. 29892146 PMC5993895

[B3] Baker A. B. Gibson W. J. Kolachalama V. B. Golomb M. Indolfi L. Spruell C. (2012). Heparanase regulates thrombosis in vascular injury and stent-induced flow disturbance. J. Am. Coll. Cardiol. 59 (17), 1551–1560. 10.1016/j.jacc.2011.11.057 22516446 PMC4191917

[B4] Barman S. A. Li X. Haigh S. Kondrikov D. Mahboubi K. Bordan Z. (2019). Galectin-3 is expressed in vascular smooth muscle cells and promotes pulmonary hypertension through changes in proliferation, apoptosis, and fibrosis. Am. J. Physiol. Lung Cell. Mol. Physiol. 316 (5), L784-L797–l797. 10.1152/ajplung.00186.2018 30724100 PMC6589585

[B5] Bellaye P. S. Yanagihara T. Granton E. Sato S. Shimbori C. Upagupta C. (2018). Macitentan reduces progression of TGF-β1-induced pulmonary fibrosis and pulmonary hypertension. Eur. Respir. J. 52 (2), 1701857. 10.1183/13993003.01857-2017 29976656

[B6] Biasin V. Wygrecka M. Bärnthaler T. Jandl K. Jain P. P. Bálint Z. (2018). Docking of meprin α to heparan sulphate protects the endothelium from inflammatory cell extravasation. Thromb. Haemost. 118 (10), 1790–1802. 10.1055/s-0038-1670657 30235485

[B7] Blich M. Golan A. Arvatz G. Sebbag A. Shafat I. Sabo E. (2013). Macrophage activation by heparanase is mediated by TLR-2 and TLR-4 and associates with plaque progression. Arterioscler. Thromb. Vasc. Biol. 33 (2), e56–e65. 10.1161/ATVBAHA.112.254961 23162016 PMC3548034

[B8] Bush M. A. Anstey N. M. Yeo T. W. Florence S. M. Granger D. L. Mwaikambo E. D. (2021). Vascular dysfunction in malaria: understanding the role of the endothelial glycocalyx. Front. Cell. Dev. Biol. 9, 751251. 10.3389/fcell.2021.751251 34858979 PMC8631294

[B9] Cassady S. J. Ramani G. V. (2020). Right heart failure in pulmonary hypertension. Cardiol. Clin. 38 (2), 243–255. 10.1016/j.ccl.2020.02.001 32284101

[B10] Chen J. Miao J. Zhou D. Liao J. Wang Z. Lin Z. (2022). Upregulation of mechanosensitive channel Piezo1 involved in high shear stress-induced pulmonary hypertension. Thromb. Res. 218, 52–63. 10.1016/j.thromres.2022.08.006 35988445

[B11] Chen S. Ye F. Chen Y. Chen Y. Zhao H. Yatsunami R. (2011). Biochemical analysis and kinetic modeling of the thermal inactivation of MBP-fused heparinase I: implications for a comprehensive thermostabilization strategy. Biotechnol. Bioeng. 108 (8), 1841–1851. 10.1002/bit.23144 21445884

[B12] Chen Y. B. (2019). Autophagy and its role in pulmonary hypertension. Aging Clin. Exp. Res. 31 (8), 1027–1033. 10.1007/s40520-018-1063-1 30406918

[B13] Cimmino G. Cirillo P. (2018). Tissue factor: newer concepts in thrombosis and its role beyond thrombosis and hemostasis. Cardiovasc Diagn Ther. 8 (5), 581–593. 10.21037/cdt.2018.10.14 30498683 PMC6232348

[B14] Crispel Y. Ghanem S. Attias J. Kogan I. Brenner B. Nadir Y. (2017). Involvement of the heparanase procoagulant domain in bleeding and wound healing. J. Thromb. Haemost. 15 (7), 1463–1472. 10.1111/jth.13707 28439967

[B15] Cruz L. A. Tellman T. V. Farach-Carson M. C. (2020). Flipping the molecular switch: influence of perlecan and its modifiers in the tumor microenvironment. Adv. Exp. Med. Biol. 1245, 133–146. 10.1007/978-3-030-40146-7_6 32266656

[B16] Cui H. Tan Y. X. Österholm C. Zhang X. Hedin U. Vlodavsky I. (2016). Heparanase expression upregulates platelet adhesion activity and thrombogenicity. Oncotarget 7 (26), 39486–39496. 10.18632/oncotarget.8960 27129145 PMC5129947

[B17] Davizon-Castillo P. Allawzi A. Sorrells M. Fisher S. Baltrunaite K. Neeves K. (2020). Platelet activation in experimental murine neonatal pulmonary hypertension. Physiol. Rep. 8 (5), e14386. 10.14814/phy2.14386 32163236 PMC7066872

[B18] Del Pozo R. Hernandez Gonzalez I. Escribano-Subias P. (2017). The prostacyclin pathway in pulmonary arterial hypertension: A clinical review. Expert Rev. Respir. Med. 11 (6), 491–503. 10.1080/17476348.2017.1317599 28399721

[B19] Deng C. Wu D. Yang M. Chen Y. Ding H. Zhong Z. (2016). The role of tissue factor and autophagy in pulmonary vascular remodeling in a rat model for chronic thromboembolic pulmonary hypertension. Respir. Res. 17 (1), 65. 10.1186/s12931-016-0383-y 27234007 PMC4884382

[B20] Ding M. Huang Z. Wang X. Liu X. Xu L. Chen P. (2022). Heparan sulfate proteoglycans-mediated targeted delivery of TGF-β1-binding peptide to liver for improved anti-liver fibrotic activity *in vitro* and *in vivo* . Int. J. Biol. Macromol. 209, 1516–1525. 10.1016/j.ijbiomac.2022.04.085 35452701

[B21] Dull R. O. Cluff M. Kingston J. Hill D. Chen H. Hoehne S. (2012). Lung heparan sulfates modulate K(fc) during increased vascular pressure: evidence for glycocalyx-mediated mechanotransduction. Am. J. Physiol. Lung Cell. Mol. Physiol. 302 (9), L816–L828. 10.1152/ajplung.00080.2011 22160307 PMC3362156

[B22] Ebong E. E. Lopez-Quintero S. V. Rizzo V. Spray D. C. Tarbell J. M. (2014). Shear-induced endothelial NOS activation and remodeling via heparan sulfate, glypican-1, and syndecan-1. Integr. Biol. (Camb) 6 (3), 338–347. 10.1039/c3ib40199e 24480876 PMC3996848

[B23] Enomoto N. Suzuki S. Hozumi H. Karayama M. Suzuki Y. Furuhashi K. (2021). Diagnostic and prognostic significance of serum angiopoietin-1 and -2 concentrations in patients with pulmonary hypertension. Sci. Rep. 11 (1), 15502. 10.1038/s41598-021-94907-w 34326408 PMC8322335

[B24] Evans C. E. Cober N. D. Dai Z. Stewart D. J. Zhao Y. Y. (2021). Endothelial cells in the pathogenesis of pulmonary arterial hypertension. Eur. Respir. J. 58 (3), 2003957. 10.1183/13993003.03957-2020 33509961 PMC8316496

[B25] Feng F. Wang L. J. Li J. C. Chen T. T. Liu L. (2023). Role of heparanase in ARDS through autophagy and exosome pathway (review). Front. Pharmacol. 14, 1200782. 10.3389/fphar.2023.1200782 37361227 PMC10285077

[B26] Florian J. A. Kosky J. R. Ainslie K. Pang Z. Dull R. O. Tarbell J. M. (2003). Heparan sulfate proteoglycan is a mechanosensor on endothelial cells. Circ. Res. 93 (10), e136–e142. 10.1161/01.RES.0000101744.47866.D5 14563712

[B27] Frangogiannis N. (2020). Transforming growth factor-β in tissue fibrosis. J. Exp. Med. 217 (3), e20190103. 10.1084/jem.20190103 32997468 PMC7062524

[B28] Fu S. Hu X. Ma Z. Wei Q. Xiang X. Li S. (2022). Unfractionated heparin attenuated histone-induced pulmonary syndecan-1 degradation in mice: a preliminary study on the roles of heparinase pathway. Inflammation 45 (2), 712–724. 10.3390/cells11040712 34657233

[B29] Galiè N. Humbert M. Vachiery J. L. Gibbs S. Lang I. Torbicki A. 2015. 2015 ESC/ERS guidelines for the diagnosis and treatment of pulmonary hypertension: the joint task force for the diagnosis and treatment of pulmonary hypertension of the European society of Cardiology (ESC) and the European respiratory society (ERS): endorsed by: association for European paediatric and congenital Cardiology (AEPC), international society for heart and lung transplantation (ISHLT). Eur. Heart J. 37(1): p. 67–119. 10.1093/eurheartj/ehv317 26320113

[B30] Gan X. Wang J. Su B. Wu D. (2011). Evidence for direct activation of mTORC2 kinase activity by phosphatidylinositol 3,4,5-trisphosphate. J. Biol. Chem. 286 (13), 10998–11002. 10.1074/jbc.M110.195016 21310961 PMC3064154

[B31] Garsen M. Lenoir O. Rops A. L. W. M. M. Dijkman H. B. Willemsen B. van Kuppevelt T. H. (2016). Endothelin-1 induces proteinuria by heparanase-mediated disruption of the glomerular glycocalyx. J. Am. Soc. Nephrol. 27 (12), 3545–3551. 10.1681/ASN.2015091070 27026367 PMC5118481

[B32] Goodall K. J. Poon I. K. H. Phipps S. Hulett M. D. (2014). Soluble heparan sulfate fragments generated by heparanase trigger the release of pro-inflammatory cytokines through TLR-4. PLoS One 9 (10), e109596. 10.1371/journal.pone.0109596 25295599 PMC4190175

[B33] Guo J. Yang Z. C. Liu Y. (2019). Attenuating pulmonary hypertension by protecting the integrity of glycocalyx in rats model of pulmonary artery hypertension. Inflammation 42 (6), 1951–1956. 10.1007/s10753-019-01055-5 31267273

[B34] Gutter-Kapon L. Alishekevitz D. Shaked Y. Li J. P. Aronheim A. Ilan N. (2016). Heparanase is required for activation and function of macrophages. Proc. Natl. Acad. Sci. U. S. A. 113 (48), E7808-E7817–e7817. 10.1073/pnas.1611380113 27849593 PMC5137702

[B35] Hao N. B. Tang B. Wang G. Z. Xie R. Hu C. J. Wang S. M. (2015). Hepatocyte growth factor (HGF) upregulates heparanase expression via the PI3K/Akt/NF-κB signaling pathway for gastric cancer metastasis. Cancer Lett. 361 (1), 57–66. 10.1016/j.canlet.2015.02.043 25727320

[B36] Henderson N. C. Rieder F. Wynn T. A. (2020). Fibrosis: from mechanisms to medicines. Nature 587 (7835), 555–566. 10.1038/s41586-020-2938-9 33239795 PMC8034822

[B37] Höök M. Wasteson A. Oldberg A. (1975). A heparan sulfate-degrading endoglycosidase from rat liver tissue. Biochem. Biophys. Res. Commun. 67 (4), 1422–1428. 10.1016/0006-291x(75)90185-0 1035

[B38] Hu G. Shao M. Gao X. Wang F. Liu C. (2017). Probing cleavage promiscuity of heparinase III towards chemoenzymatically synthetic heparan sulfate oligosaccharides. Carbohydr. Polym. 173, 276–285. 10.1016/j.carbpol.2017.05.071 28732866

[B39] Hu P. Xu Y. Jiang Y. Huang J. Liu Y. Wang D. (2022). The mechanism of the imbalance between proliferation and ferroptosis in pulmonary artery smooth muscle cells based on the activation of SLC7A11. Eur. J. Pharmacol. 928, 175093. 10.1016/j.ejphar.2022.175093 35700835

[B40] Humbert M. Kovacs G. Hoeper M. M. Badagliacca R. Berger R. M. F. Brida M. 2022. 2022 ESC/ERS Guidelines for the diagnosis and treatment of pulmonary hypertension. Eur. Heart J. 43(38): p. 3618–3731. 10.1093/eurheartj/ehac237 36017548

[B41] Hurst L. A. Dunmore B. J. Long L. Crosby A. Al-Lamki R. Deighton J. (2017). TNFα drives pulmonary arterial hypertension by suppressing the BMP type-II receptor and altering NOTCH signalling. Nat. Commun. 8, 14079. 10.1038/ncomms14079 28084316 PMC5241886

[B42] Kadenhe-Chiweshe A. Papa J. McCrudden K. W. Frischer J. Bae J. O. Huang J. (2008). Sustained VEGF blockade results in microenvironmental sequestration of VEGF by tumors and persistent VEGF receptor-2 activation. Mol. Cancer Res. 6 (1), 1–9. 10.1158/1541-7786.MCR-07-0101 18234958

[B43] Koganti R. Suryawanshi R. Shukla D. (2020). Heparanase, cell signaling, and viral infections. Cell. Mol. Life Sci. 77 (24), 5059–5077. 10.1007/s00018-020-03559-y 32462405 PMC7252873

[B44] Konstam M. A. Kiernan M. S. Bernstein D. Bozkurt B. Jacob M. Kapur N. K. (2018). Evaluation and management of right-sided heart failure: A scientific statement from the American heart association. Circulation 137 (20), e578–e622. 10.1161/CIR.0000000000000560 29650544

[B45] Lázár Z. Mészáros M. Bikov A. (2020). The nitric oxide pathway in pulmonary arterial hypertension: pathomechanism, biomarkers and drug targets. Curr. Med. Chem. 27 (42), 7168–7188. 10.2174/0929867327666200522215047 32442078

[B46] Lee C. Mitsialis S. A. Aslam M. Vitali S. H. Vergadi E. Konstantinou G. (2012). Exosomes mediate the cytoprotective action of mesenchymal stromal cells on hypoxia-induced pulmonary hypertension. Circulation 126 (22), 2601–2611. 10.1161/CIRCULATIONAHA.112.114173 23114789 PMC3979353

[B47] Lee C. S. Zhai Y. Shang R. Wong T. Mattison A. J. Cen H. H. (2022). Flow-induced secretion of endothelial heparanase regulates cardiac lipoprotein lipase and changes following diabetes. J. Am. Heart Assoc. 11 (23), e027958. 10.1161/JAHA.122.027958 36416172 PMC9851453

[B48] Lei Q. Yu Z. Li H. Cheng J. Wang Y. (2022). Fatty acid-binding protein 5 aggravates pulmonary artery fibrosis in pulmonary hypertension secondary to left heart disease via activating wnt/β-catenin pathway. J. Adv. Res. 40, 197–206. 10.1016/j.jare.2021.11.011 36100327 PMC9481948

[B49] Lerner I. Hermano E. Zcharia E. Rodkin D. Bulvik R. Doviner V. (2011). Heparanase powers a chronic inflammatory circuit that promotes colitis-associated tumorigenesis in mice. J. Clin. Investig. 121 (5), 1709–1721. 10.1172/JCI43792 21490396 PMC3083784

[B50] Levy-Adam F. Feld S. Cohen-Kaplan V. Shteingauz A. Gross M. Arvatz G. (2010). Heparanase 2 interacts with heparan sulfate with high affinity and inhibits heparanase activity. J. Biol. Chem. 285 (36), 28010–28019. 10.1074/jbc.M110.116384 20576607 PMC2934666

[B51] Li J. Cao F. Yin H. L. Huang Z. J. Lin Z. T. Mao N. (2020). Ferroptosis: past, present and future. Cell. Death Dis. 11 (2), 88. 10.1038/s41419-020-2298-2 32015325 PMC6997353

[B52] Li J. Zhang X. Lu Z. Yu S. P. Wei L. (2012). Expression of heparanase in vascular cells and astrocytes of the mouse brain after focal cerebral ischemia. Brain Res. 1433, 137–144. 10.1016/j.brainres.2011.11.032 22169133 PMC3286644

[B53] Li Y. Ren W. Wang X. Yu X. Cui L. Li X. (2019). MicroRNA-150 relieves vascular remodeling and fibrosis in hypoxia-induced pulmonary hypertension. Biomed. Pharmacother. 109, 1740–1749. 10.1016/j.biopha.2018.11.058 30551428

[B54] Liao Y. E. Liu J. Arnold K. (2023). Heparan sulfates and heparan sulfate binding proteins in sepsis. Front. Mol. Biosci. 10, 1146685. 10.3389/fmolb.2023.1146685 36865384 PMC9971734

[B55] Lindahl U. Li J. P. (2020). Heparanase - discovery and targets. Adv. Exp. Med. Biol. 1221, 61–69. 10.1007/978-3-030-34521-1_2 32274706

[B56] Liu J. Wang W. Wang L. Chen S. Tian B. Huang K. (2018). IL-33 initiates vascular remodelling in hypoxic pulmonary hypertension by up-regulating HIF-1α and VEGF expression in vascular endothelial cells. EBioMedicine 33, 196–210. 10.1016/j.ebiom.2018.06.003 29921553 PMC6085568

[B57] Lucena S. V. Moura G. E. D. D. Rodrigues T. Watashi C. M. Melo F. H. Icimoto M. Y. (2018). Heparan sulfate proteoglycan deficiency up-regulates the intracellular production of nitric oxide in Chinese hamster ovary cell lines. J. Cell. Physiol. 233 (4), 3176–3194. 10.1002/jcp.26160 28833096

[B58] Lukasz A. Hillgruber C. Oberleithner H. Kusche-Vihrog K. Pavenstädt H. Rovas A. (2017). Endothelial glycocalyx breakdown is mediated by angiopoietin-2. Cardiovasc Res. 113 (6), 671–680. 10.1093/cvr/cvx023 28453727

[B59] Lv Q. Wu K. Liu F. Wu W. Chen Y. Zhang W. (2018). Interleukin-17A and heparanase promote angiogenesis and cell proliferation and invasion in cervical cancer. Int. J. Oncol. 53 (4), 1809–1817. 10.3892/ijo.2018.4503 30066843

[B60] Lv Q. Zeng J. He L. (2016). The advancements of heparanase in fibrosis. Int. J. Mol. Epidemiol. Genet. 7 (4), 137–140. 28078057 PMC5218871

[B61] Masola V. Granata S. Bellin G. Gambaro G. Onisto M. Rugiu C. (2017). Specific heparanase inhibition reverses glucose-induced mesothelial-to-mesenchymal transition. Nephrol. Dial. Transpl. 32 (7), 1145–1154. 10.1093/ndt/gfw403 28064160

[B62] Mizumura K. Cloonan S. Choi M. E. Hashimoto S. Nakahira K. Ryter S. W. Autophagy: friend or foe in lung disease? Ann. Am. Thorac. Soc., 2016. 13 Suppl. 1(Suppl. 1): p. S40–S47. 10.1513/AnnalsATS.201507-450MG 27027951 PMC5466160

[B63] Nadir Y. Brenner B. (2012). Heparanase procoagulant activity. Thromb. Res. 129 (Suppl. 1), S76–S79. 10.1016/S0049-3848(12)70021-X 22682139

[B64] Nadir Y. Brenner B. (2010). Heparanase procoagulant effects and inhibition by heparins. Thromb. Res. 125 (Suppl. 2), S72–S76. 10.1016/S0049-3848(10)70018-9 20434010

[B65] Nadir Y. (2014). Heparanase and coagulation-new insights. Rambam Maimonides Med. J. 5 (4), e0031. 10.5041/RMMJ.10165 25386347 PMC4222420

[B66] Nadir Y. (2020). Heparanase in the coagulation system. Adv. Exp. Med. Biol. 1221, 771–784. 10.1007/978-3-030-34521-1_33 32274737

[B67] Pandolfi R. Barreira B. Moreno E. Lara-Acedo V. Morales-Cano D. Martínez-Ramas A. (2017). Role of acid sphingomyelinase and IL-6 as mediators of endotoxin-induced pulmonary vascular dysfunction. Thorax 72 (5), 460–471. 10.1136/thoraxjnl-2015-208067 27701117

[B68] Peled E. Assalia M. Axelman L. Norman D. Nadir Y. (2016). PO-49 - bone microinfarction and microcirculation thrombosis; is it a possible mechanism for bone pain among cancer patients? Thromb. Res. 140 (Suppl. 1), S194–S195. 10.1016/S0049-3848(16)30182-7 27161735

[B69] Poch D. Mandel J. (2021). Pulmonary hypertension. Ann. Intern Med. 174 (4), Itc49–itc64. 10.7326/AITC202104200 33844574

[B70] Potje S. R. Costa T. J. Fraga-Silva T. F. C. Martins R. B. Benatti M. N. Almado C. E. L. (2021). Heparin prevents *in vitro* glycocalyx shedding induced by plasma from COVID-19 patients. Life Sci. 276, 119376. 10.1016/j.lfs.2021.119376 33781826 PMC7997864

[B71] Riaz A. Ilan N. Vlodavsky I. Li J. P. Johansson S. (2013). Characterization of heparanase-induced phosphatidylinositol 3-kinase-AKT activation and its integrin dependence. J. Biol. Chem. 288 (17), 12366–12375. 10.1074/jbc.M112.435172 23504323 PMC3636920

[B72] Rivara S. Milazzo F. M. Giannini G. (2016). Heparanase: A rainbow pharmacological target associated to multiple pathologies including rare diseases. Future Med. Chem. 8 (6), 647–680. 10.4155/fmc-2016-0012 27057774

[B73] Roucourt B. Meeussen S. Bao J. Zimmermann P. David G. (2015). Heparanase activates the syndecan-syntenin-ALIX exosome pathway. Cell. Res. 25 (4), 412–428. 10.1038/cr.2015.29 25732677 PMC4387558

[B74] Samokhin A. O. Stephens T. Wertheim B. M. Wang R. S. Vargas S. O. Yung L. M. (2018). NEDD9 targets COL3A1 to promote endothelial fibrosis and pulmonary arterial hypertension. Sci. Transl. Med. 10 (445), eaap7294. 10.1126/scitranslmed.aap7294 29899023 PMC6223025

[B75] Sanderson R. D. Elkin M. Rapraeger A. C. Ilan N. Vlodavsky I. (2017). Heparanase regulation of cancer, autophagy and inflammation: new mechanisms and targets for therapy. Febs J. 284 (1), 42–55. 10.1111/febs.13932 27758044 PMC5226874

[B76] Schmidt E. P. Yang Y. Janssen W. J. Gandjeva A. Perez M. J. Barthel L. (2012). The pulmonary endothelial glycocalyx regulates neutrophil adhesion and lung injury during experimental sepsis. Nat. Med. 18 (8), 1217–1223. 10.1038/nm.2843 22820644 PMC3723751

[B77] Secchi M. F. Masola V. Zaza G. Lupo A. Gambaro G. Onisto M. (2015). Recent data concerning heparanase: focus on fibrosis, inflammation and cancer. Biomol. Concepts 6 (5-6), 415–421. 10.1515/bmc-2015-0021 26552066

[B78] Seyfarth H. J. Sack U. Gessner C. Wirtz H. (2015). Angiogenin, bFGF and VEGF: angiogenic markers in breath condensate of patients with pulmonary hypertension. Pneumologie 69 (4), 207–211. 10.1055/s-0034-1391775 25853270

[B79] Shteingauz A. Boyango I. Naroditsky I. Hammond E. Gruber M. Doweck I. (2015). Heparanase enhances tumor growth and chemoresistance by promoting autophagy. Cancer Res. 75 (18), 3946–3957. 10.1158/0008-5472.CAN-15-0037 26249176 PMC4573896

[B80] Simonneau G. Montani D. Celermajer D. S. Denton C. P. Gatzoulis M. A. Krowka M. (2019). Haemodynamic definitions and updated clinical classification of pulmonary hypertension. Eur. Respir. J. 53 (1), 1801913. 10.1183/13993003.01913-2018 30545968 PMC6351336

[B81] Soon E. Holmes A. M. Treacy C. M. Doughty N. J. Southgate L. Machado R. D. (2010). Elevated levels of inflammatory cytokines predict survival in idiopathic and familial pulmonary arterial hypertension. Circulation 122 (9), 920–927. 10.1161/CIRCULATIONAHA.109.933762 20713898

[B82] Stoler-Barak L. Petrovich E. Aychek T. Gurevich I. Tal O. Hatzav M. (2015). Heparanase of murine effector lymphocytes and neutrophils is not required for their diapedesis into sites of inflammation. Faseb J. 29 (5), 2010–2021. 10.1096/fj.14-265447 25634957

[B83] Suzuki A. Tomita H. Okada H. (2022). Form follows function: the endothelial glycocalyx. Transl. Res. 247, 158–167. 10.1016/j.trsl.2022.03.014 35421613

[B84] Thenappan T. Ormiston M. L. Ryan J. J. Archer S. L. (2018). Pulmonary arterial hypertension: pathogenesis and clinical management. Bmj 360, j5492. 10.1136/bmj.j5492 29540357 PMC6889979

[B85] Thompson C. A. Purushothaman A. Ramani V. C. Vlodavsky I. Sanderson R. D. (2013). Heparanase regulates secretion, composition, and function of tumor cell-derived exosomes. J. Biol. Chem. 288 (14), 10093–10099. 10.1074/jbc.C112.444562 23430739 PMC3617250

[B86] Tournier A. Wahl D. Chaouat A. Max J. P. Regnault V. Lecompte T. (2010). Calibrated automated thrombography demonstrates hypercoagulability in patients with idiopathic pulmonary arterial hypertension. Thromb. Res. 126 (6), e418–e422. 10.1016/j.thromres.2010.08.020 20888030

[B87] Udjus C. Cero F. T. Halvorsen B. Behmen D. Carlson C. R. Bendiksen B. A. (2019). Caspase-1 induces smooth muscle cell growth in hypoxia-induced pulmonary hypertension. Am. J. Physiol. Lung Cell. Mol. Physiol. 316 (6), L999-L1012–l1012. 10.1152/ajplung.00322.2018 30908936

[B88] Vlodavsky I. Barash U. Nguyen H. M. Yang S. M. Ilan N. (2021). Biology of the heparanase-heparan sulfate Axis and its role in disease pathogenesis. Semin. Thromb. Hemost. 47 (3), 240–253. 10.1055/s-0041-1725066 33794549 PMC9097616

[B89] Vlodavsky I. Friedmann Y. Elkin M. Aingorn H. Atzmon R. Ishai-Michaeli R. (1999). Mammalian heparanase: gene cloning, expression and function in tumor progression and metastasis. Nat. Med. 5 (7), 793–802. 10.1038/10518 10395325

[B90] Vlodavsky I. Gross-Cohen M. Weissmann M. Ilan N. Sanderson R. D. (2018). Opposing functions of heparanase-1 and heparanase-2 in cancer progression. Trends Biochem. Sci. 43 (1), 18–31. 10.1016/j.tibs.2017.10.007 29162390 PMC5741533

[B91] Vlodavsky I. Singh P. Boyango I. Gutter-Kapon L. Elkin M. Sanderson R. D. (2016). Heparanase: from basic research to therapeutic applications in cancer and inflammation. Drug Resist Updat 29, 54–75. 10.1016/j.drup.2016.10.001 27912844 PMC5447241

[B92] Wang Z. Chen J. Babicheva A. Jain P. P. Rodriguez M. Ayon R. J. (2021). Endothelial upregulation of mechanosensitive channel Piezo1 in pulmonary hypertension. Am. J. Physiol. Cell. Physiol. 321 (6), C1010–c1027. 10.1152/ajpcell.00147.2021 34669509 PMC8714987

[B93] Waxman A. Restrepo-Jaramillo R. Thenappan T. Ravichandran A. Engel P. Bajwa A. (2021). Inhaled treprostinil in pulmonary hypertension due to interstitial lung disease. N. Engl. J. Med. 384 (4), 325–334. 10.1056/NEJMoa2008470 33440084

[B94] White T. A. Witt T. A. Pan S. Mueske C. S. Kleppe L. S. Holroyd E. W. (2010). Tissue factor pathway inhibitor overexpression inhibits hypoxia-induced pulmonary hypertension. Am. J. Respir. Cell. Mol. Biol. 43 (1), 35–45. 10.1165/rcmb.2009-0144OC 19648471 PMC2911569

[B95] Xiang J. Lu M. Shi M. Cheng X. Kwakwa K. A. Davis J. L. (2022). Heparanase blockade as a novel dual-targeting therapy for COVID-19. J. Virol. 96 (7), e0005722. 10.1128/jvi.00057-22 35319225 PMC9006938

[B96] Xiao Z. Zhao W. Yang B. Zhang Z. Guan H. Linhardt R. J. (2011). Heparinase 1 selectivity for the 3,6-di-O-sulfo-2-deoxy-2-sulfamido-alpha-D-glucopyranose (1,4) 2-O-sulfo-alpha-L-idopyranosyluronic acid (GlcNS3S6S-IdoA2S) linkages. Glycobiology 21 (1), 13–22. 10.1093/glycob/cwq123 20729345 PMC2998982

[B97] Xie S. S. Deng Y. Guo S. L. Li J. Q. Zhou Y. C. Liao J. (2022). Endothelial cell ferroptosis mediates monocrotaline-induced pulmonary hypertension in rats by modulating NLRP3 inflammasome activation. Sci. Rep. 12 (1), 3056. 10.1038/s41598-022-06848-7 35197507 PMC8866506

[B98] Yang M. Tang B. Wang S. Tang L. Wen D. Vlodavsky I. (2022). Non-enzymatic heparanase enhances gastric tumor proliferation via TFEB-dependent autophagy. Oncogenesis 11 (1), 49. 10.1038/s41389-022-00424-4 35970822 PMC9378687

[B99] Yang W. J. Zhang G. L. Cao K. X. Liu X. N. Wang X. M. Yu M. W. (2020). Heparanase from triple-negative breast cancer and platelets acts as an enhancer of metastasis. Int. J. Oncol. 57 (4), 890–904. 10.3892/ijo.2020.5115 32945393 PMC7473754

[B100] Yen W. Cai B. Yang J. Zhang L. Zeng M. Tarbell J. M. (2015). Endothelial surface glycocalyx can regulate flow-induced nitric oxide production in microvessels *in vivo* . PLoS One 10 (1), e0117133. 10.1371/journal.pone.0117133 25575016 PMC4289188

[B101] Yuan F. Yang Y. Zhou H. Quan J. Liu C. Wang Y. (2022). Heparanase in cancer progression: structure, substrate recognition and therapeutic potential. Front. Chem. 10, 926353. 10.3389/fchem.2022.926353 36157032 PMC9500389

[B102] Zetser A. Bashenko Y. Edovitsky E. Levy-Adam F. Vlodavsky I. Ilan N. (2006). Heparanase induces vascular endothelial growth factor expression: correlation with p38 phosphorylation levels and Src activation. Cancer Res. 66 (3), 1455–1463. 10.1158/0008-5472.CAN-05-1811 16452201

[B103] Zhang J. Yang J. m. Wang H. j. Ru G. q. Fan D. m. (2013). Synthesized multiple antigenic polypeptide vaccine based on B-cell epitopes of human heparanase could elicit a potent antimetastatic effect on human hepatocellular carcinoma *in vivo* . PLoS One 8 (1), e52940. 10.1371/journal.pone.0052940 23308126 PMC3538634

[B104] Zhang J. Yang J. Zuo T. Ma S. Xokrat N. Hu Z. (2021). Heparanase-driven sequential released nanoparticles for ferroptosis and tumor microenvironment modulations synergism in breast cancer therapy. Biomaterials 266, 120429. 10.1016/j.biomaterials.2020.120429 33035717

[B105] Zhang M. Xin W. Yu Y. Yang X. Ma C. Zhang H. (2020a). Programmed death-ligand 1 triggers PASMCs pyroptosis and pulmonary vascular fibrosis in pulmonary hypertension. J. Mol. Cell. Cardiol. 138, 23–33. 10.1016/j.yjmcc.2019.10.008 31733200

[B106] Zhang S. Liu X. Ge L. L. Li K. Sun Y. Wang F. (2020b). Mesenchymal stromal cell-derived exosomes improve pulmonary hypertension through inhibition of pulmonary vascular remodeling. Respir. Res. 21 (1), 71. 10.1186/s12931-020-1331-4 32192495 PMC7082982

[B107] Zhang T. He X. Caldwell L. Goru S. K. Ulloa Severino L. Tolosa M. F. (2022). NUAK1 promotes organ fibrosis via YAP and TGF-β/SMAD signaling. Sci. Transl. Med. 14 (637), eaaz4028. 10.1126/scitranslmed.aaz4028 35320001

[B108] Zhang Y. Xu C. B. (2020). The roles of endothelin and its receptors in cigarette smoke-associated pulmonary hypertension with chronic lung disease. Pathol. Res. Pract. 216 (9), 153083. 10.1016/j.prp.2020.153083 32825951

[B109] Zhu S. Li J. Loka R. S. Song Z. Vlodavsky I. Zhang K. (2020). Modulating heparanase activity: tuning sulfation pattern and glycosidic linkage of oligosaccharides. J. Med. Chem. 63 (8), 4227–4255. 10.1021/acs.jmedchem.0c00156 32216347 PMC7376576

[B110] Zolty R. (2020). Pulmonary arterial hypertension specific therapy: the old and the new. Pharmacol. Ther. 214, 107576. 10.1016/j.pharmthera.2020.107576 32417272

